# Locally-advanced unresected uterine leiomyosarcoma with triple-modality treatment combining radiotherapy, chemotherapy and hyperthermia: A case report

**DOI:** 10.3892/ol.2014.2193

**Published:** 2014-05-28

**Authors:** AYA SHIRAFUJI, AKIKO SHINAGAWA, TETSUJI KUROKAWA, YOSHIO YOSHIDA

**Affiliations:** 1 Department of Obstetrics and Gynecology, Faculty of Medical Sciences, University of Fukui, Fukui, Japan; 2 Department of Obstetrics and Gynecology, Fukui Aiiku Hospital, Fukui, Japan

**Keywords:** advanced leiomyosarcoma, radiation therapy, chemotherapy, hyperthermia

## Abstract

Advanced uterine leiomyosarcoma (LMS) is a rare and extremely aggressive disease. In patients with advanced and unresected uterine LMS, multidisciplinary therapy is the best treatment option, although no consensus exists on the efficacy of the treatment. The present study describes the case of a 41-year-old female who underwent laparotomy due to a large uterine tumor. Exploratory laparotomy revealed a large tumor that had extended from the pelvic wall to the outside of the pelvis and then invaded the colon. Large residual tumors remained present in the pelvis following suboptimal debulking surgery. Subsequent to surgery, the patient was treated with adjuvant radiotherapy, followed by chemotherapy with regional whole pelvis hyperthermia (HT). Computed tomography revealed stable disease prior and subsequent to combination treatment. While treatment was being administered for third/fourth-degree burns and subcutaneous fatty necrosis, the patient developed multi-organ failure and succumbed. The present case report describes the potential for using a combination of chemotherapy, HT and radiotherapy in patients with LMS. The development of an effective protocol is required for the administration of chemotherapy, HT and radiotherapy in patients with advanced unresected LMS.

## Introduction

Uterine leiomyosarcoma (LMS) is a rare and extremely aggressive disease. In patients with advanced LMS, the five-year survival rate is <20%. In addition, suboptimal primary surgery that induces tumor injury and a cut-through of the uterine sarcoma is associated with a worse prognosis compared with complete resection of the uterine sarcoma (1,2).

In patients with advanced and unresected uterine LMS, multidisciplinary therapy is the best treatment option for the extension of life. For treatment using chemotherapy subsequent to radiotherapy, numerous agents have been investigated in patients with LMS, however, results have been unsatisfactory (Table I) (3–16). A number of studies have shown that chemotherapy-based treatments may have potential in patients with LMS through the simultaneous use of hyperthermia (HT) and drugs (17–20). However, to the best of our knowledge, there have been no studies on the treatment with radiotherapy followed by a combination of chemotherapy and HT in patients with locally-advanced, unresected uterine LMS. To the best of our knowledge, the present study is the first to report the use of these three modalities for the treatment of locally-advanced, unresected uterine LMS.

## Case report

### Patient presentation

A 41-year old female (gravida 2, para 2) presented to the University of Fukui Hospital (Fukui, Japan) with prolonged uterine bleeding and lower abdominal fullness. The patient had previously been treated for a large symptomatic leiomyoma with monthly injections of a long-acting gonadotropin-releasing hormone (GnRH) agonist at the Fukui Aiiku Hospital (Fukui, Japan). Upon examination, the patient appeared anemic and had a palpable abdominal mass that was the same size as the head of a newborn child. Magnetic resonance imaging revealed a large, heterogeneously hyperintense mass in the T1- and T2-weighted images of the pelvic cavity. Despite these abnormal findings, the patient selected conservative treatment in order to preserve fertility and due to the previous good response to GnRH agonist treatment. The patient was administered monthly injections of 1.88 mg leuprolide acetate. Prior to the third injection, an increase in the size of the uterine mass was observed, and two weeks after the injection the patient presented with abdominal pain, bleeding and leg edema. The patient subsequently decided to undergo surgery.

### Surgery

**Figure 1:**
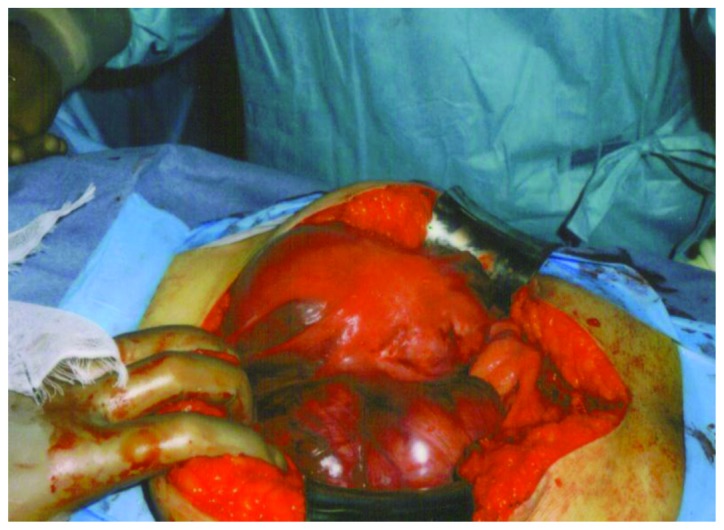
Exploratory laparotomy revealed a large tumor extending outside of the pelvis and invading the colon.

**Figure 2:**
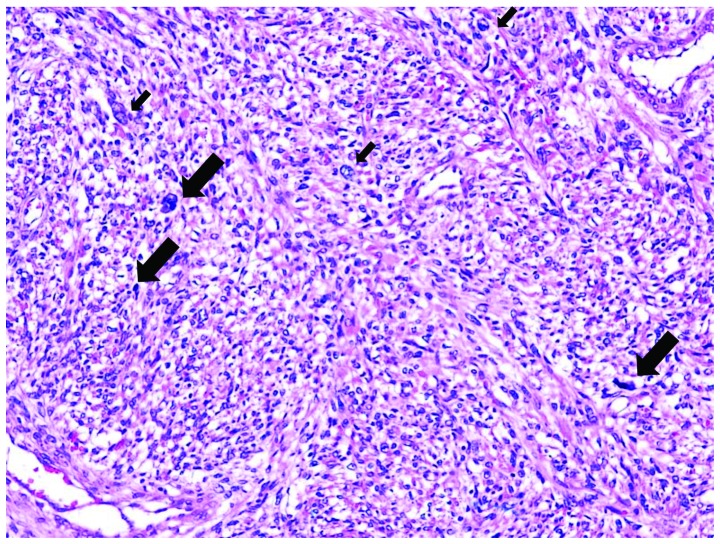
Surgical specimen showing smooth muscle cells with nuclear atypia (small arrows) and a high mitotic index, with >10 mitotic figures per high power field (large arrows). Hematoxylin and eosin staining; magnification, ×20.

Exploratory laparotomy revealed a large tumor that had extended from the pelvic wall to the outside of the pelvis and then invaded the colon (Fig. 1). Suboptimal debulking was performed, with a supra-hysterectomy and resection of the metastatic masses. Large residual tumors remained in the pelvis. Microscopically, the mass was composed of an LMS component with nuclear atypia and a high mitotic index (Fig. 2) and a leiomyoma component.

### Adjuvant chemotherapy and radiotherapy

Following suboptimal debulking surgery, the patient presented with lower abdominal pain and continuous vaginal bleeding. A previous study showed that radiotherapy improves the local status of uterine sarcomas (21), thus, subsequent to obtaining informed consent, adjuvant radiotherapy was administered, followed by chemotherapy at the University of Fukui Hospital. The patient received 15-MV external-beam radiotherapy to the whole pelvis at a dose of 50.4 Gy, in five fractions of 1.8 Gy per week, using a four-field box technique according to our institutional protocol. Computed tomography (CT) revealed stable disease (SD) according to the Response Evaluation Criteria in Solid Tumors (RECIST), prior and subsequent to radiotherapy. Two weeks after radiotherapy, the patient received intravenous gemcitabine (900 mg/m^2^ administered over 90 min on days 1 and 8) and docetaxel (75 mg/m^2^ on day 8) with granulocyte growth factor support on day 9 of a 21-day cycle to be scheduled every three weeks. Based on the results of the previous radiotherapy (15), a 25% lower dose of docetaxel was administered. Following three months of gemcitabine and docetaxel chemotherapy, the patient was hospitalized due to grade II pulmonary toxicity. The patient was treated with antibiotics and showed marked improvement. Subsequent to a further three months of receiving the same chemotherapy, the patient was readmitted with grade II pulmonary toxicity. The patient was again treated with antibiotics and showed marked improvement. A CT scan revealed the SD status of the LMS according to the RECIST, prior and subsequent to the six months of chemotherapy.

### HT treatment

Based on the patient’s tumor response, toxicity and performance status, HT was added to the chemotherapy regimen subsequent to obtaining informed consent. Regional whole pelvis HT was administered in the same week that the patient received gemcitabine (900 mg/m^2^ administered over 90 min) and docetaxel (75 mg/m^2^). Thermometry catheters were placed in the rectum, bladder and vagina for thermal dose calculations. Following appropriate adjustments to the treatment settings, the power output was increased until the patient’s tolerance threshold was reached. HT treatment was administered for 60 min after vaginal measurements had reached 40°C. Subsequent to 6 weeks of HT with chemotherapy, the patient was hospitalized with grade III burns and subcutaneous fatty necrosis. Therefore, HT treatment was withdrawn. CT revealed SD of the LMS according to the RECIST, prior and subsequent to six months of receiving the combination of HT and chemotherapy. While treatment was being administered for the grade III/IV burns and subcutaneous fatty necrosis, the patient developed multi-organ failure and succumbed.

## Discussion

Adujuvant pelvic radiotherapy may improve local pelvic control, but dose not improve patient survival (21). Thus, in patients with symptomatic (for example exhibiting vaginal bleeding), unresected or advanced LMS, radiotherapy is optimal.

In patients with advanced or unresected LMS, systemic treatment is the best option for the extension of life. Table I shows an overview of the clinical trials of chemotherapy followed by radiotherapy used to treat patients with advanced LMS in previous studies. Gemcitabine and docetaxel are the most common agents used for non-primary chemotherapy in patients with recurrent and advanced uterine sarcoma. Hensley *et al* (15) performed a phase II trial of gemcitabine and docetaxel in patients with unresectable LMS. The study included patients who had received prior pelvic radiation, those who had become worse following doxorubicin-based therapy and those who had not received prior chemotherapy. A 25% lower dose of the two agents was administered to the patients who had received prior pelvic radiation. A complete response or a partial response (PR) were observed in 53% of the enrolled patients. Hematological toxicity was common, while neutropenic fever and bleeding were rare (15). In the present case, the patient received 25% lower doses of gemcitabine and docetaxel, as the patient had received prior pelvic radiation treatment. Although PR was not observed and the side-effects were acceptable, for patients who have undergone prior treatment, particularly radiotherapy, the use of 25% lower doses of gemcitabine and docetaxel should be considered.

Research into LMS continues to focus on the identification of active drugs and treatment combinations. A number of studies have shown the potential for a response to chemotherapy by the simultaneous use of HT and drugs (17–20). Wiedemann *et al* (17) reported that whole body hyperthermia (WBH) may enhance the therapeutic index of specific chemotherapeutic agents, such as ifosfamide, carboplatin and etoposide (ICE), resulting in a response rate of 63% (17). However, Pereira Arias *et al* (18) reported the development of an acute systemic inflammatory response syndrome with multiple organ dysfunction syndrome following administration of WBH in combination with ICE. If chemotherapic agents and hyperthemia are selected appropriately, for example, changing WBH to whole-pelvic HT, HT with chemotherapy may be a safe and useful procedure for the treatment of locally-advanced cervical cancer. Westerman *et al* (19) reported that the combination of full-dose radiotherapy, chemotherapy and HT is a feasible and effective treatment strategy for patients with advanced cervical carcinoma without concessions to radiotherapy, chemotherapy or HT dose, compared with patients receiving single- or combined-modality treatment. Additionally, Mohamed *et al* (20) reported that HT increased the cytotoxicity of docetaxel and gemcitabine in mouse fibrosarcoma. Thus, in the present study, a compounding effect was expected with the combination of docetaxel, gemcitabine and HT for the patient with unresected LMS who had undergone prior radiotherapy. In the present case, this combination had moderate efficacy. The cytotoxicity of these drugs is synergized by heat, but the timing between the chemotherapy and HT may not have been adequate for the present patient. For the concomitant use of radiotherapy, chemotherapy and HT for the treatment of patients with cervical carcinoma, Westermann *et al* (19) advised that chemotherapy and HT should be administered concurrently, preferentially 1 h, but no more than 6 h, prior to radiotherapy. The optimal sequence of the administration of these three therapeutic modalities for the treatment of LMS has yet to be elucidated. The analysis of an effective protocol is required for the administration of chemotherapy, HT and radiotherapy in patients with unresected LMS.

In the present case, the combination of HT with chemotherapy was effective, however, grade III/IV burns and subcutaneous fatty necrosis toxicity occurred. At present, no consensus exists on the efficacy of treatment for locally-advanced, unresected uterine LMS. The present case study described the potential for a combination treatment using chemotherapy, HT and radiotherapy. In conclusion, further investigation is required into an effective protocol for the administration of chemotherapy, HT and radiotherapy in patients with unresected LMS.

## Figures and Tables

**Table I:** Review of the schedules of chemotherapy followed by radiotherapy used to treat patients with leiomyosarcoma of the uterus in previous studies.

A, Single-agent				

Drug	A/B	Schedule	Response rate, %	First author (year) [ref]
Gemcitabine	20/31	1,250 mg/m^2^ on days 1 and 8 in a 3-weekly schedule	3.23	Svancarova L *et al* (2002) [3]
	15/29	1,250 mg/m^2^ every week × three, cycles repeated every 28 days	3	Okuno S *et al* (2002) [4]
Paclitaxel	15/48	135 mg/m^2^ for patients with prior radiotherapy every 3 weeks	8.4	Gallup DG *et al* (2003) [5]
Trabectedin	21/36	A 24-h continuous iv infusion at a dose of 1.5 g/m^2^ every 3 weeks	8	Garcia-Carbonera R *et al* (2004) [6]
Trimetrexate	7/23	5 mg/m^2^/day orally for 5 days every other week	4.3	Smith HO *et al* (2002) [7]
Etoposide	6/29	30–40 mg/m^2^/day for prior radiotherapy as a single dose for 21 days, every 28 days	6.9	Rose PG *et al* (1998) [8]
	7/28	100 mg/m^2^ orally ond ays 1,3 and 5	11	Slayton RE *et al* (1987) [9]
Amonafide	8/26	300 mg/m^2^ × 5 days every 3 weeks	4	Asbury R *et al* (1998) [10]

B, Combination				

Drug	A/B	Schedule	Response rate, %	First author (year) [ref]

Dacarbazine + Mitimycin + Doxorubicine + Cisplatin	7/18	Day 0 consisting of dacarbazine 750 mg/m^2^ iv over 2 h, mitomycin 6 mg/m^2^ iv over 2–5 min, doxorubicin 40 mg/m^2^ iv over 2–5 min and cisplatin 60 mg/m^2^ iv over 2 h, retreated at 4-week intervals	27.8	Long HJ III *et al* (2005) [11]
Mitimycin + Doxorubicine + Cisplatin	8/35	Mitomycin 8 mg/m^2^ and doxorubicin 40 mg/m^2^ each by iv injection, followed by cisplatin 60 mg/m^2^ by 2-h iv at 3-week intervals	23	Edmonson JH *et al* (2002) [12]
Hydroxyurea + Dacarbazine + Etoposide	11/32	Hydroxyurea 2 g in divided doses on day 1, 700 mg/m^2^ dacarbazine and 100 mg/m^2^ etoposide on day 2 and 100 mg/m^2^ etoposide on days 3 and 4	18	Currie J *et al* (1996) [13]
Ifosfamide + Doxorubicin	9/34	Ifosfamide, 5.0 g/m^2^/24 h, and mesna, 6.0 g/m^2^/36 h, by continuous iv infusion preceded by doxorubicin, 50 mg/m^2^ iv over 15 min Each course of therapy was repeated every 3 weeks	30.3	Sutton G *et al* (1996) [14]
Gemcitabine + Docetaxel	14/34	Gemcitabine 900 mg/m^2^ iv on days 1 and 8 plus docetaxel 100 mg/m^2^ iv on day 8 delivered every 21 days (25% lower doses if prior radiotherapy)	53	Hensley ML *et al* (2002) [15]
Gemcitabine + Docetaxel	17/39	Gemcitabine 900 mg/m^2^ iv on days 1 and 8 plus docetaxel 100 mg/m^2^ iv on day 8 delivered every 21 days	27	Hensley ML *et al* (2008) [16]

A/B, number of patients prior to radiotherapy/number of all patients; iv, intravenous.
